# A Novel 5-Enolpyruvylshikimate-3-Phosphate Synthase Shows High Glyphosate Tolerance in *Escherichia coli* and Tobacco Plants

**DOI:** 10.1371/journal.pone.0038718

**Published:** 2012-06-08

**Authors:** Gaoyi Cao, Yunjun Liu, Shengxue Zhang, Xuewen Yang, Rongrong Chen, Yuwen Zhang, Wei Lu, Yan Liu, Jianhua Wang, Min Lin, Guoying Wang

**Affiliations:** 1 College of Agriculture and Biotechnology, China Agricultural University, Beijing, China; 2 Institute of Crop Sciences, Chinese Academy of Agricultural Sciences, Beijing, China; 3 Biotechnology Research Institute, Chinese Academy of Agricultural Sciences, Beijing, China; Universidad Nacional Autonoma de Mexico, Instituto de Biotecnologia, Mexico

## Abstract

A key enzyme in the shikimate pathway, 5-enolpyruvylshikimate-3-phosphate synthase (EPSPS) is the primary target of the broad-spectrum herbicide glyphosate. Identification of new *aroA* genes coding for EPSPS with a high level of glyphosate tolerance is essential for the development of glyphosate-tolerant crops. In the present study, the glyphosate tolerance of five bacterial *aroA* genes was evaluated in the *E. coli aroA*-defective strain ER2799 and in transgenic tobacco plants. All five *aroA* genes could complement the *aroA*-defective strain ER2799, and *AM79 aroA* showed the highest glyphosate tolerance. Although glyphosate treatment inhibited the growth of both WT and transgenic tobacco plants, transgenic plants expressing *AM79 aroA* tolerated higher concentration of glyphosate and had a higher fresh weight and survival rate than plants expressing other *aroA* genes. When treated with high concentration of glyphosate, lower shikimate content was detected in the leaves of transgenic plants expressing *AM79 aroA* than transgenic plants expressing other *aroA* genes. These results suggest that *AM79 aroA* could be a good candidate for the development of transgenic glyphosate-tolerant crops.

## Introduction

Glyphosate (N-phosphonomethyl glycine), an important and potent herbicide, is widely used to control weeds in agricultural fields. Glyphosate inhibits the enzyme 5-enolpyruvylshikimate-3-phosphate synthase (EPSPS;EC 2.5.1.19), which converts phosphoenolpyruvate (PEP) and shikimate-3-phosphate (S3P) to 5-enolpyruvylshikimate-3-phosphate (EPSP) and shuts down the shikimate pathway, leading to plant death. Two types of EPSP synthases have been classified [1]. Type I EPSP synthases have been identified mainly in plants and bacteria, and type II EPSP synthases have been identified in some forms of bacteria. Type I EPSP synthases are naturally sensitive to glyphosate, whereas type II EPSP synthases are tolerant of glyphosate [2]–[4]. After the primary target of glyphosate was identified as EPSPS in the 1980s [5], EPSPS became the top choice for the development of transgenic glyphosate-tolerant crops. However, over-expression of most wild EPSPS in transgenic plants can not confer plants with glyphosate tolerance [6].

Mutagenesis of EPSPS is one way to obtain glyphosate-tolerant EPSPS. It was first reported that the expression of an altered EPSPS confers resistance to the herbicide glyphosate [7]. Expression of the *Salmonella typhimurium* EPSPS mutant (Pro101 to Ser) in transgenic tobacco plants confers tolerance to glyphosate [8]. Mutagenesis and structure analysis have revealed the mechanism of EPSPS function [4], [9]–[12]. A single Ala residue at position 100 leads to the CP4 EPSPS (obtained from *Agrobacterium* sp. strain CP4) becoming insensitive to glyphosate, while natural plant and bacterial enzymes share a highly conserved Gly residue at this position [4]. Double mutation (T97I, P101S) of type I EPSPS cause the shift of the Gly residue at position 96 toward the glyphosate binding site, leading to glyphosate tolerance [12]. A P106L mutant of rice EPSPS was selected based on the directed evolution strategy and conferred high glyphosate tolerance in *E. coli* and in transgenic tobacco plants [13]. The change of two codons provides the maize EPSPS with glyphosate tolerance [14]. A proline-to-serine substitution at position 106 in the goosegrass's predicted EPSPS mature protein coding region produces a five fold higher glyphosate tolerance capability than the sensitive biotype [15].

In *E. coli*, EPSPS is encoded by *aroA*. Glyphosate-tolerant EPSPS can also be obtained by screening the bacteria grown in a glyphosate-contamination environment. Organisms can survive in chemically stressed environments through physiological adaptation resulting from modifications of gene expression or through adaptive mutation to relieve selective pressure [16]–[18]. In recent years, a number of glyphosate-resistant *aroA* genes have been cloned from bacteria [19]–[21]. However, only one of the type II EPSPS genes from *Agrobacterium* sp. CP4 and the point mutations of class I EPSPS from *E. coli* or maize have been successfully used in commercial transgenic crops [4].

In our previous work, several bacterial *aroA* genes (*G2 aroA*, *HTG7 aroA*, *A1501 aroA*, *RD aroA* and *AM79 aroA*), were cloned from *Pseudomonas fluorescens* [22], *Halomonas variabilis* [23], *Pseudomonas stutzeri* [24], and uncultured soil bacteria [25]–[26], respectively. Here, we evaluated the glyphosate tolerance of these five *aroA* genes in *E. coli* and in transgenic tobacco plants. Our results showed that *AM79 aroA* is potentially a better candidate for development of transgenic glyphosate-tolerant crops than other *aroA* genes.

## Results

### Evaluation of five *aroA* genes in the *aroA*-deficient *E. coli* mutant

**Figure 1:**
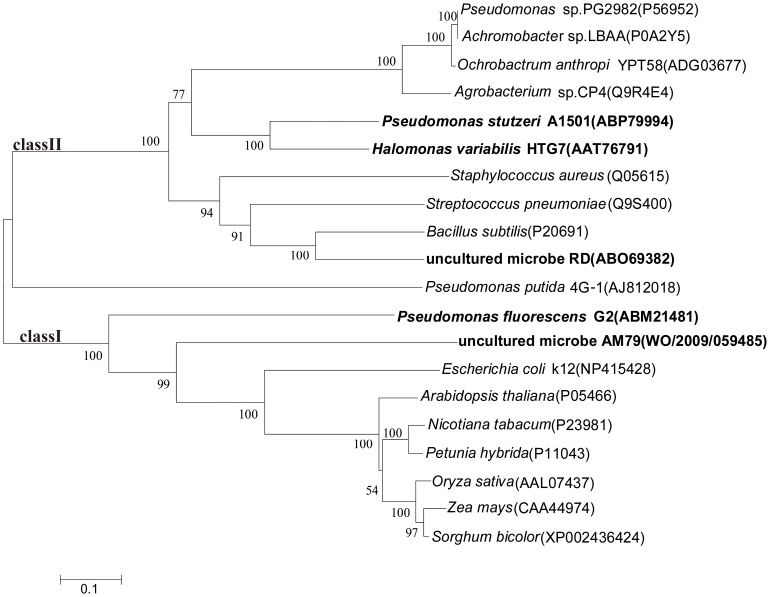
Phylogenetic analysis of the five EPSPS and related proteins. The phylogenetic tree was based on homologous sequences of the EPSPS proteins and the neighbor-joining methods (MEGA4.0). The percentage of the tree from 1000 bootstrap resamples supporting the topology is indicated when above 50. Accession numbers or international patent publication numbers are shown in parentheses. The scale bar represents 0.1 substitutions per position.

Five *aroA* genes, named as *G2 aroA*, *HTG7 aroA*, *A1501 aroA*, *RD aroA* and *AM79 aroA*, were cloned from the bacteria grown in soil heavily contaminated with a high concentration of glyphosate [22]–[25]. An alignment analysis of these five EPSPS proteins with some other known EPSPS proteins showed that G2 EPSPS and AM79 EPSPS belong to class I, while HTG7 EPSPS, A1501 EPSPS and RD EPSPS belong to class II EPSPS (Figure 1).

**Figure 2:**
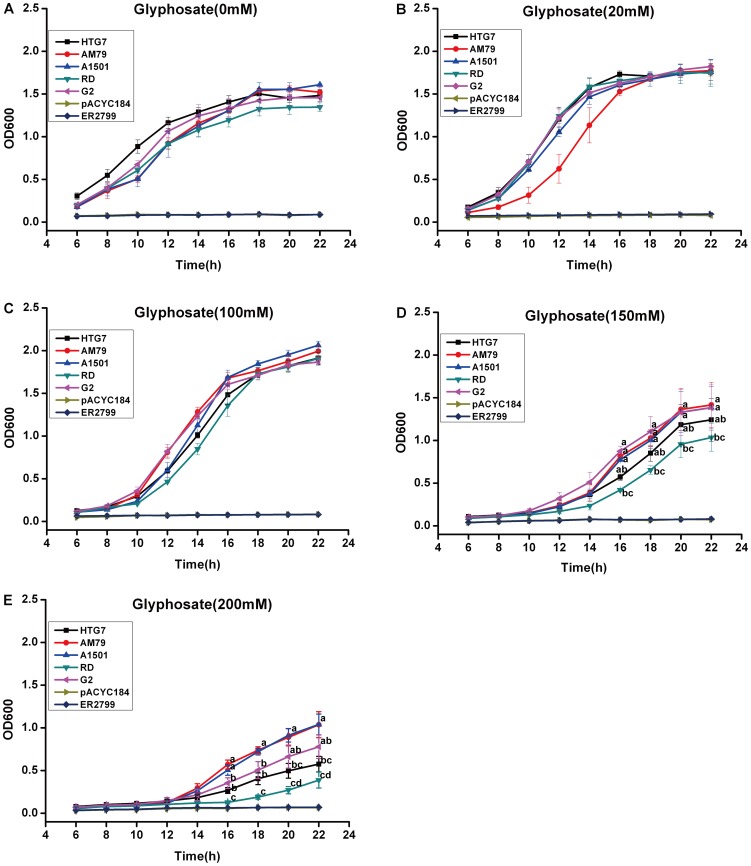
Glyphosate tolerance of *E. coli* containing five *aroA* genes. The plasmids pACYC184, pACYC-HTG7, pACYC-AM79, pACYC-A1501, pACYC-RD and pACYC-G2 were transformed into *E. coli* ER2799 competent cells for growth curve measurement. M9 liquid medium was supplemented with different concentrations of glyphosate. OD_600_ was recorded every two hours starting 6 h after treatment. Data are shown as the average ± S.E. of three independent experiments. Experimental data was tested by ANOVA analysis and different letter means significant difference at *P*<0.05 level. (A) Growth curve of ER2799 and the strain harboring different plasmids under 0 mM glyphosate. (B) Growth curve of ER2799 and the strain harboring different plasmids under 20 mM glyphosate. (C) Growth curve of ER2799 and the strain harboring different plasmids under 100 mM glyphosate. (D) Growth curve of ER2799 and the strain harboring different plasmids under 150 mM glyphosate. (E) Growth curve of ER2799 and the strain harboring different plasmids under 200 mM glyphosate.

To evaluate the glyphosate tolerance, these five genes were cloned into the *Eco*RI site of the low-copy plasmid pACYC184 and transformed into the *E. coli* strain ER2799, which is a stable *aroA*-defective mutant that cannot grow in minimal medium. The ER2799 containing different *aroA* genes could grow in the minimal M9 liquid medium with different concentrations of glyphosate. In the M9 medium containing 20 or 100 mM glyphosate, all strains expressing *aroA* genes grew well. When the glyphosate concentration was increased to 150 mM and 200 mM, strains expressing *RD aroA* and *HTG7 aroA* grew slower than the bacteria transformed with the other three *aroA* genes, indicating a lower glyphosate tolerance of *RD aroA* and *HTG7 aroA*. Although similar transcript levels were detected in strains expressing these *aroA* (Figure S1), strains expressing *AM79 aroA* and *A1501 aroA* showed significantly higher glyphosate tolerance than others (Figure 2), suggesting that the difference in glyphosate tolerance among strains expressing these *aroA* was due to the enzyme itself.

### Comparson of enzyme kinetic parameters among the five bacterial EPSPS

Five *aroA* genes were cloned into pET-28a expression vector and the expressed proteins in *E. coli* were purified for enzyme assay. AM79 EPSPS and RD EPSPS had lower *K*_m_ values (14.59 and 7.34), indicating they had higher PEP affinities than other EPSPS proteins. A1501 EPSPS and AM79 EPSPS had higher *K*_i_ values than other three EPSPS proteins, meaning that they can tolerate high glyphosate concentrations (Table 1; Figure S2). Since glyphosate competes with PEP for binding in the active site of EPSPS, high *K*_i_/*K*_m_ is critical for EPSPS to maintain enzymatic activity in the presence of glyphosate inhibitor. AM79 EPSPS had the highest *K*_i_/*K*_m_ value (10.6) among the five EPSPS (Table 1), indicating *AM79 aroA* is a good candidate gene for transgenic crops.

**Table 1:** Kinetic parameters of five EPSPS enzymes

	*V*_max_(U mg^−1^)	*K*_m_ (PEP; µM)	*K*_i_ (glyphosate; µM)	*K*_i_/*K*_m_
HTG7	55.80±3.04	39.06±2.69	74.11±10.89	1.9
AM79	48.62±0.36	14.59±0.11	154.39±4.06	10.6
A1501	16.67±1.58	92.42±22.07	467.35±30.21	5.1
RD	94.08±4.67	7.34±1.97	28.83±4.50	3.9
G2	38.16±2.58	49.35±2.68	44.71±5.20	0.9

Data was shown as mean ± S.D. of three independent experiments.

### Transgenic tobacco seedlings over-expressing AM79 aroA showed higher glyphosate tolerance

**Figure 3:**
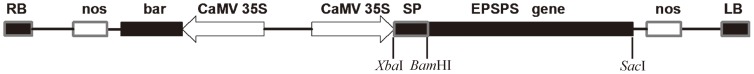
T-DNA cassette containing *aroA* gene for plant transformation. LB and RB, left and right border of T-DNA; bar, phosphinothricin acetyltransferase gene; nos, *NOS* terminator; CaMV 35S, cauliflower mosaic virus 35S promoter; SP, coding sequence of signal peptide of pea rib-1,5-bisphospate carboxylase (*rbcS*) small subunit; EPSPS gene, five microorganism *aroA* gene.

To investigate whether *AM79 aroA* is preferable to the development of glyphosate-resistant transgenic crops, we assessed the glyphosate tolerance conferred by the five *aroA* genes in transgenic tobacco plants. The signal peptide sequence of the pea rib-1,5-bisphospate carboxylase (*rbcS*) small subunit was fused in front of the *aroA* gene with the correct reading frame to direct the EPSPS to the plant chloroplast. The signal peptide and the EPSPS were controlled by the CaMV 35S promoter and cloned into the plasmid pCAMBIA3301 to construct the vectors for plant transformation (Figure 3). The vectors were introduced into tobacco plants (*Nicotiana tabacum* var. *Samsum*) via *Agrobacterium*-mediated transformation.

**Figure 4:**
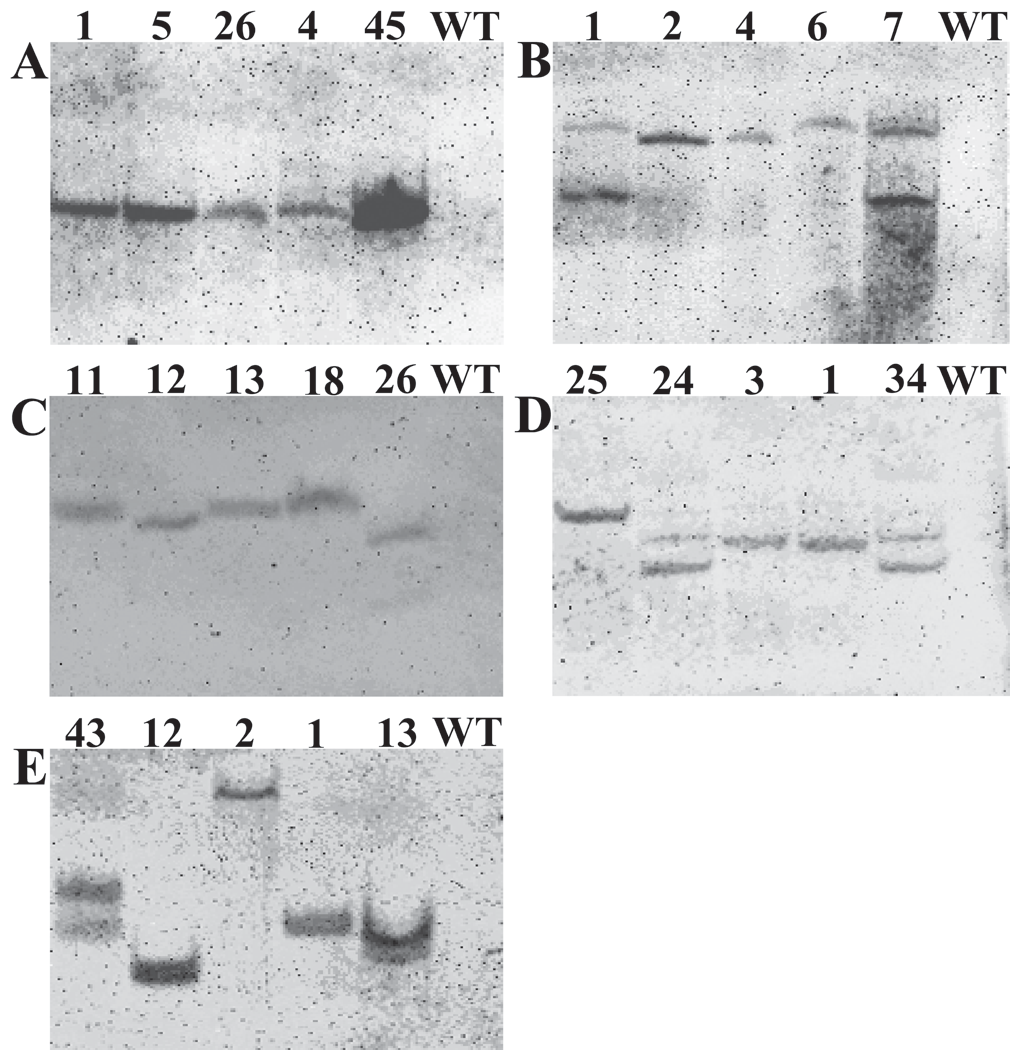
Southern blot analysis of transgenic tobacco plants. (A) HTG7; (B) AM79; (C) A1501; (D) RD; (E) G2. Five transgenic lines each construct were analyzed. 100 µg genomic DNA was digested with *Hin*dIII which has only one site in the plasmid, so the band numbers are equal to the copy number of transgene.

Tobacco T1 progeny seeds were germinated on MS medium without glyphosate using 10 mg L^−1^ phosphinothricin as a selective reagent to eliminate non-transgenic plant. The transgenic plants were confirmed by PCR amplification (Figure S3) and Southern blot analysis, which showed that most transgenic lines have one or two copy of transgenes (Figure 4). Our results also showed that the signal peptide did improve the glyphosate tolerance conferred by the *G2 aroA* in transgenic tobacco (Figure S4).

**Figure 5:**
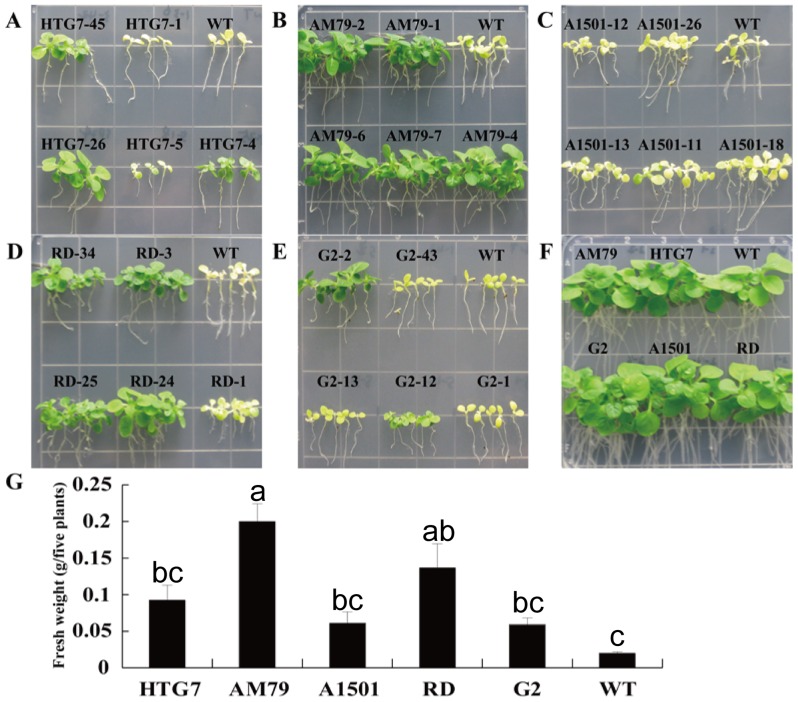
Glyphosate tolerance of transgenic tobacco seedlings on plates containing 1 mM glyphosate. T1 tobacco seeds were germinated on the MS medium containing 10 mg L^−1^ PPT and grown for 7 days. The live seedlings were transferred to MS medium on plates containing 1 mM glyphosate. Photographs were taken two weeks later, and the fresh weight was measured at the same time. (A) Photograph of tobacco harboring *HTG7 aroA* and WT grown on the medium containing 1 mM glyphosate. (B) Photograph of tobacco harboring *AM79 aroA* and WT grown on the medium containing 1 mM glyphosate. (C) Photograph of tobacco harboring *A1501 aroA* and WT grown on the medium containing 1 mM glyphosate. (D) Photograph of tobacco harboring *RD aroA* and WT grown on the medium containing 1 mM glyphosate. (E) Photograph of tobacco harboring *G2 aroA* and WT grown on the medium containing 1 mM glyphosate. (F) Photograph of tobacco plants grown on the medium without glyphosate. (G) Fresh weight of the tobacco plants. Data are shown as the average ± S.E. of seven to ten independent transgenic lines. Experimental data was tested by ANOVA analysis and different letter in each column means significant difference at *P*<0.05 level.

Seven to ten independent transgenic lines for each gene were chosen to assess glyphosate tolerance. Transgenic tobacco seedlings were grown vertically on the MS medium containing 1 mM glyphosate for fifteen days. On the medium without glyphosate, all of the transgenic plants grew similarly to the WT plants (Figure 5F). Treatment with 1 mM glyphosate killed the WT plants and inhibited the growth of the transgenic plants over-expressing the *aroA* gene (Figure 5). The transgenic plants expressing different *aroA* genes showed a different response to the 1 mM glyphosate treatment. Plants over-expressing *AM79 aroA* grew more quickly than the plants expressing other *aroA* genes, and their leaves remained green 15 days after treatment with 1 mM glyphosate.

The fresh weight of seven to ten independent transgenic lines for each gene was measured to assess the inhibition of plant growth by glyphosate. The significant difference was observed among transgenic plants expressing various *aroA*, and transgenic plants expressing *AM79 aroA* had the highest fresh weight (Figure 5). These results clearly show that the over-expression of *AM79 aroA* in plants offers higher glyphosate tolerance than other genes. We further chose five transgenic lines for each construct to investigate the transcription of *aroA* genes using quantitative real-time RT-PCR. Relative lower transcription level occurred for *A1501 aroA* and *G2 aroA* compared with other three *aroA* genes, might due to their higher GC content. Different transcription levels were observed among the transgenic lines expressing the same *aroA* (Figure S5), and the transcription level was correlated with the glyphosate tolerance of transgenic lines (data not shown).

**Figure 6:**
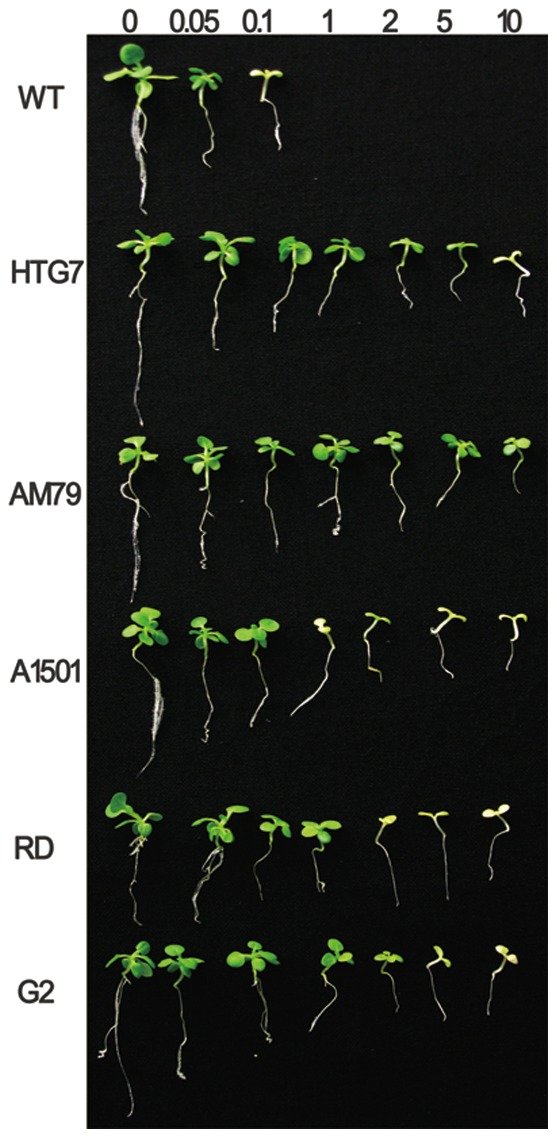
Glyphosate tolerance of transgenic tobacco under different glyphosate concentrations. Tobacco seeds of generation T1 were germinated on the MS medium containing 10 mg L^−1^ PPT and grown for 7 days at 100 µmol s^−1^ m^−2^ with a 16 h light/8 h dark period. The live seedlings were transferred to MS medium in plates containing different amounts of glyphosate and grown vertically for another two weeks.

The glyphosate tolerance of WT tobacco and transgenic seedlings were also assessed at various concentrations (Figure 6). The results showed that WT plants were only able to tolerate 0.1 mM glyphosate or less. However, transgenic tobacco seedlings expressing different *aroA* genes could tolerate a much higher concentrations of glyphosate. Compared to transgenic lines expressing other *aroA*, tobacco plants over-expressing *AM79 aroA* were more tolerant and could keep green leaves on the medium containing 10 mM glyphosate (Figure 6).

### Transgenic tobacco plants over-expressing AM79 aroA grown in a greenhouse showed higher glyphosate tolerance

**Figure 7:**
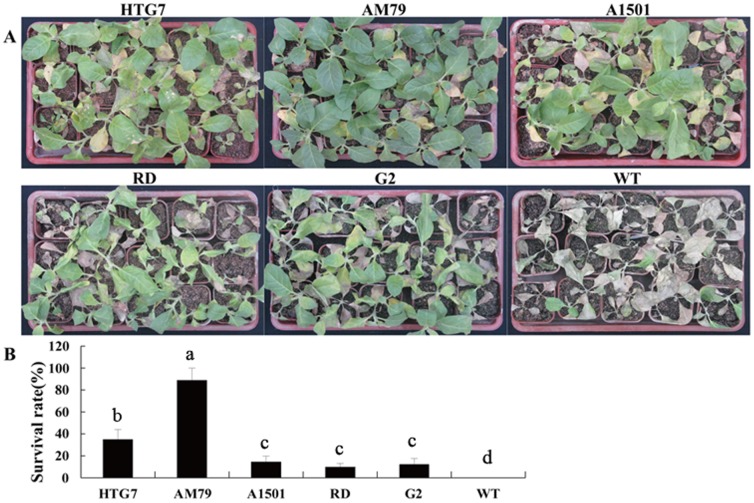
Glyphosate tolerance of transgenic tobacco plants in a greenhouse when sprayed with 6 L ha^−1^ Roundup®. T1 tobacco seeds were germinated on the MS medium containing 10 mg L^−1^ PPT and grown for 7 days at 100 µmol s^−1^ m^−2^ with a 16 h light/8 h dark period. The live seedlings were transferred to soil in pots and grown for another month. Six-to eight-leaf stage transgenic plants were sprayed with 6 L ha^−1^ Roundup®. Two weeks after treatment, injury was observed, and survival rate was measured. (A) Photograph of tobacco plants two weeks after glyphosate treatment. (B) Survival rate of tobacco plants. Data are shown as the average ± S.E. of seven to ten independent transgenic lines. Experimental data was tested by ANOVA analysis and different letter in each column means significant difference at *P*<0.05 level.

To further confirm the glyphosate tolerance of transgenic plants, six-to-eight-leaf stage transgenic tobacco plants grown in a greenhouse were sprayed with Roundup® at an equal dose of 6 L ha^−1^. Fifteen days after treatment, all WT tobacco plants died, whereas some transgenic plants remained living (Figure 7A). A survival rate of only approximately 10% was observed for transgenic plants expressing *A1501 aroA*, *RD aroA* and *G2 aroA*. Transgenic plants expressing *HTG7 aroA* or *AM79 aroA* had survival rates of 35.01% and 88.75%, respectively. ANOVA analysis showed significant difference among transgenic lines (Figure 7B). These results confirm that the over-expression of *AM79 aroA* can provide plants with a high glyphosate tolerance.

### Transgenic plants over-expressing AM79 aroA had lower shikimate accumulation after glyphosate treatment

**Figure 8:**
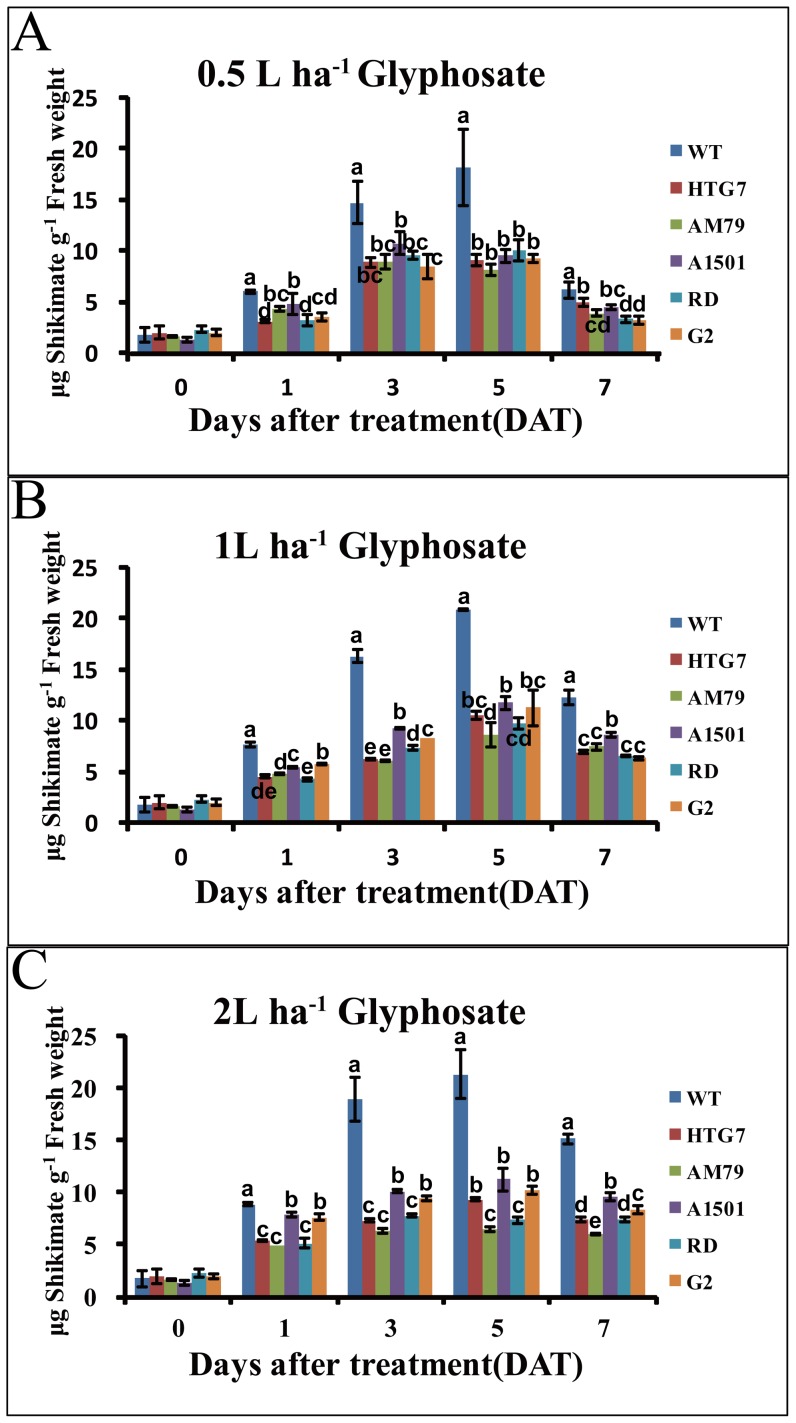
Shikimate content in the leaves of transgenic tobacco plants sprayed with different amounts of glyphosate. One-month-old plants grown in a green house were sprayed with 0.5, 1 or 2 L ha-1 Roundup®. Leaf samples were taken at 0, 1, 3, 5 and 7 d after glyphosate treatment for shikimate measurement. Data are shown as the average ±S.E. of three independent experiments. Experimental data was tested by ANOVA analysis and different letter in each column means significant difference at P<0.05 level.

Glyphosate shuts down the shikimate pathway by inhibiting the EPSPS enzymes that convert PEP and S3P to EPSP. Glyphosate treatment increases the shikimate content and is thus an indicator of plant tolerance to glyphosate [27]. Tobacco plants were sprayed with 0.5, 1, or 2 L ha^−1^ Roundup®, and the shikimate content in the leaves were measured after 0, 1, 3, 5 and 7 d of treatment. Before treatment, WT and transgenic plants had similar shikimate contents. Treatments with different amounts of glyphosate all increased the shikimate contents in the leaves of WT and transgenic plants, reaching a maximum on the fifth day. However, significantly more shikimate contents occurred in WT leaves than that in transgenic plant leaves (Figure 8). When treated with high glyphosate concentration (2 L ha^−1^), the lowest shikimate accumulation occurred in the transgenic plants expressing *AM79 aroA*, compared with transgenic plants expressing other *aroA* genes, indicating AM79 EPSPS can confer transgenic plants with high glyphosate tolerance.

## Discussion

EPSPS were chosen as the first choice to develop transgenic glyphosate-tolerant crops because they are the primary target of glyphosate [5]. Since the 1980s, researchers have begun to isolate glyphosate-insensitive EPSPS from bacteria or plants, and numerous promising enzymes have been identified by microbial screening and selective evolution [4], [13], [28], [29]. The *Agrobacterium* sp. strain CP4 is a naturally occurring, glyphosate-tolerant microbe in environments contaminated with high concentration of glyphosate, and the CP4 EPSPS has been commercially used in genetically modified crops [30]. Some other types of EPSPS were identified from other bacteria species such as *Streptococcus pneumonia* [31], *Ochrobactrum anthropi* [21], *Pseudomonas* sp. PG2982 [32] and *Staphylococcus aureus* [20].

Five *aroA* genes (*G2 aroA*, *HTG7 aroA*, *A1501 aroA*, *RD aroA* and *AM79 aroA*) have been isolated from highly glyphosate-tolerant bacterial strains [22]–[26]. Because the glyphosate tolerance of the native bacteria cannot mirror the tolerance of EPSPS itself, these five genes were cloned into the *Eco*RI site of the low-copy plasmid pACYC184 and transformed into the *aroA* mutant *E. coli* strain ER2799 to evaluate their glyphosate tolerance. Under low concentrations of glyphosate, all bacteria transformed with *aroA* genes showed tolerance to glyphosate, and their tolerant levels were similar. When the glyphosate concentration increased to 150 mM and 200 mM, *E. coli* strains containing *RD aroA* and *HTG7 aroA* grew slower than strains containing other three *aroA* genes, indicating a lower glyphosate tolerance of *RD aroA* and *HTG7 aroA* (Figure 2D, E). Similar transcript level of *aroA* were detected in different strains, suggesting that the different degree of glyphosate tolerance of individual EPSPS was due to the enzyme itself but not due to the different transcript level. At 200 mM glyphosate concentration, *AM79 aroA* and *A1501 aroA* showed more glyphosate tolerance than other *aroA* genes. We further compared AM79 EPSPS with the commercially used CP4 EPSPS from *Agrobacterium* sp. strain CP4. Both genes were cloned into the *Bam*HI/*Sal*I site of the low-copy plasmid pACYC184 and transformed into the *E. coli* strain ER2799. The results showed that AM79 EPSPS could provide glyphosate tolerance as effective as CP4 EPSPS (Figure S6).

The native bacterial strains tolerated high concentrations of glyphosate. The *Halomonas variabilis* strain HTG7 grew quickly on MOPS agar containing 300 mM glyphosate [23]; however, the *E. coli* strain ER2799 containing *HTG7 aroA* did not grow well under the 200 mM glyphosate condition. In the original microbial strain, other genes may also be involved in glyphosate tolerance by hydrolysis, acetylation, and oxidative cleavage of glyphosate. The glyphosate N-acetyltransferase (*GAT*) gene and the glyphosate oxidoreductase (*GOX*) gene could both take part in these pathways [33], [34]. Therefore, evaluation of the tolerance level in the same system (e.g. in the *E. coli* strain ER2799) is a better means to evaluate the glyphosate tolerance of individual *aroA* genes from different strains.

*K*_i_ value of EPSPS enzyme reflects the affinity of EPSPS binding glyphosate, and high *K*_i_/*K*_m_ is critical for EPSPS to maintain enzymatic activity in the presence of glyphosate inhibitor. For the *E. coli aroA* with T97I/P101S double mutation, which has been used to develop glyphosate-tolerant crops, its *K*_i_/*K*_m_ value is 24 [12]. CP4 EPSPS, which has also been used in glyphosate-tolerant maize and soybean, also has high *K*_i_/*K*_m_ value which is 32 [4]. AM79 EPSPS had the highest *K*_i_/*K*_m_ value (10.6) among the five EPSPS enzymes (Table 1), indicating *AM79 aroA* is a good candidate gene to develop transgenic crops. A1501 EPSPS had the highest *K*_i_ value (467.35) among the five EPSPS enzymes, indicating that it can tolerate high glyphosate concentrations. However, A1501 EPSPS had lower *K*_i_/*K*_m_ value (5.1) than AM79 EPSPS, which might be the reason that the glyphosate tolerance of transgenic plants over-expressing *A1501 aroA* was not as good as the transgenic plants over-expressing *AM79 aroA*.

It has been shown that the *G2 aroA* gene can confer a high tolerance to glyphosate in transgenic tobacco plants [22]. To investigate which *aroA* gene is the most suitable for the development of glyphosate resistant transgenic crops, we assessed the glyphosate tolerance of five genes in transgenic tobacco plants. Because EPSPS are located in chloroplasts [35], a signal peptide was added in front of EPSPS to traffic it to the chloroplast. Our results showed that including a signal peptide did improve the glyphosate tolerance plants expressing the *G2 aroA* gene in transgenic tobacco compared to that without a signal peptide (Figure S4). Integration of the *aroA* gene into the chloroplast genome is another way to locate the EPSPS in the chloroplast and to provide transgenic plants with glyphosate tolerance, and it has been shown that the *CP4 aroA* gene that was integrated in the chloroplast genome and expressed in chloroplast provided a high level of glyphosate tolerance in tobacco [36].

Glyphosate can inhibit plant growth by shutting down the shikimate pathway [15], [27]. The WT tobacco plants only tolerated 0.05 mM glyphosate. 0.1 mM glyphosate caused the WT plants to turn yellow, and concentrations higher than 0.1 mM could kill the WT plants (Figure 6). These findings are consistent with the results of other reports [13], [22]. It has also been shown that transgenic tobacco plants expressing *G2 aroA* can tolerate 1 mM glyphosate [22], and our results with new transgenic lines confirmed this fact and indicated that the transgenic plants can tolerate up to 2 mM glyphosate. The glyphosate tolerance of plants expressing the other four *aroA* genes has not been evaluated in transgenic tobacco plants before. Here, we show that tobacco plants expressing *AM79 aroA* can grow well in medium containing 10 mM glyphosate, whereas for transgenic plants expressing the other four genes, 10 mM glyphosate leads to yellow leaves and shorter roots. These results indicate that *AM79 aroA* can provide transgenic plants with a higher glyphosate tolerance than the other four *aroA* genes can. The above conclusion was confirmed by the result that transgenic plants expressing *AM79 aroA* had the highest fresh weight after 15 days of growth on the medium containing 1 mM glyphosate, and also by the result that the transgenic plants expressing *AM79 aroA* had the highest survival rate after being sprayed with 6 L ha^−1^ Roundup® in a greenhouse.

Copy numbers of transgene in transgenic plants were investigated and the results showed that most transgenic plants had one or two copies (Figure 4). Even though some transgenic lines containing the same gene all have a single copy of transgene, different transcription level was observed (Figure S5), might due to the different insertion site in the genome, leading to the different glyphosate tolerance level of these lines. Different genes have similar transcription level, except that relative lower transcription level occurred for *A1501 aroA* and *G2 aroA* compared with other three *aroA* genes, which might be due to higher gene GC content of *A1501 aroA* (64.47% GC content) and *G2 aroA* (64.49% GC content). It have been reported that GC content is positively associated with transcript abundance [37], and the bacterial *cbnA* gene was not expressed in tobacco BY-2 cells under the control of 35S promoter, due to the high 65% GC content [38].

Treatment of plants with glyphosate inhibits EPSPS activity and leads to the accumulation of shikimate [1], [39]. It has been observed that glyphosate treatment causes the accumulation of shikimate in glyphosate-insensitive tobacco cells, while there is a lack of shikimate accumulation in tolerant cells [27]. Glyphosate causes a marked increase of shikimate in both the control and tolerant alfalfa cell lines, but the accumulation of shikimate is lower in tolerant calli [40]. Consistent with above reports, treatment with a different concentration of glyphosate induced the accumulation of shikimate in WT and transgenic tobacco plants in this study. However, lower shikimate accumulation in transgenic plants over-expressing *AM79 aroA* indicated their higher glyphosate tolerance than the plants xpressing other *aroA* genes (Figure 8).

To better understand the possible mechanisms for the good glyphosate tolerance of AM79 EPSPS, we conducted sequence analysis (Figure S7). Amino acids 90–104 are strictly conserved in class I EPSPS [12]. In this region, the normal active site exists in the site 106 (number according to *E. coli* EPSPS) of class I EPSPS is Pro while is Leu is type II EPSPS. However, the residue equivalent of this site in AM79 EPSPS is Phe114 (Figure S7). Glu354 in the CP4 EPSPS amino acid sequence plays an important role in the EPSPS catalytic action for its binding capacity in the complex with the substrate [4]. This active site preserved uniformity in all the EPSPS in the equivalent regions, but AM79 EPSPS has a His356 close to Glu357 before the active site, different from with the other EPSPS enzymes (Figure S7). Glyphosate binds EPSPS in the active site by occupying the PEP-binding site, causing the inhibition of EPSPS activity. Some reports revealed that certain adjacent amino acids beside the active site can drive a shift of the EPSPS structure, producing a higher glyphosate tolerance [12]. The special amino acids Ala107 and Phe114, and the unique amino acids Ala355, His356 in AM79 EPSPS, may take part in a similar function by inhibiting the bind of glyphosate to EPSPS. This structure basis might contribute to the superior glyphosate tolerance of AM79 EPSPS.

In summary, we evaluated the glyphosate tolerance of five bacteria *aroA* genes in *E. coli* and transgenic tobacco plants and confirmed that *AM79 aroA* showed a higher glyphosate tolerance than that conferred by the other four *aroA* genes, indicating that *AM79 aroA* is a good candidate for the development of herbicide-tolerant crops. The transformation of *AM79 aroA* to crops such as maize and soybean is underway.

## Materials and Methods

### Materials

The *E. coli* strain ER2799 (with the *aroA* deleted in its genome) [41] was from the Biotechnology Research Institute, Chinese Academy of Agricultural Sciences. *Agrobacterium tumefaciens* LBA4404, plasmid pCAMBIA3301, and wild-type tobacco plants (*Nicotiana tabacum* var. *Samsum*) were preserved in our laboratory. The low-copy plasmid pACYC184, T4 DNA ligase, and restriction enzymes were obtained from New England Biolabs.

### Plasmid construction

Five *aroA* genes were amplified by PCR with the *Eco*RI site added on both sides of the gene and cloned into the *Eco*RI site of the pACYC184 plasmid to construct the vectors pACYC-HTG7, pACYC-AM79, pACYC-A1501, pACYC-RD and pACYC-G2. *CP4 aroA* gene (GenBank AB209952) was artificially synthesized and cloned into the *Bam*HI/*Sal*I site of the low-copy plasmid pACYC184 and transformed into the *E. coli* strain ER2799. All primers used in the research are listed in Table S1.

To express and purify the EPSPS proteins, five *aroA* genes were amplified by PCR adding *Bam*HI and *Hin*dIII at the 5′ end and 3′ end, respectively. The stop codon of each gene was knockout by PCR process for enhancing the binding capability by using two His tags. The five *aroA* genes were digested with *Bam*HI and *Hin*dIII and cloned into pET-28a (Novagen) digested with the same enzymes. The constructed plasmids were transformed into strain Rosetta (DE3).

To construct the plant transformation vectors, a 35S-gus-nos fragment digested from pBI121 with *Hin*dIII and *Eco*RI was cloned into the same site of pCAMBIA3301, and then the *gus* was replaced with a *G2 aroA* fragment digested with *Bam*HI and *Sac*I to construct the vector p3301-121G2. The signal peptide of the pea rib-1,5-bisphospate carboxylase (*rbcS*) small subunit was amplified using the PCR method, and *Xba*I and *Bam*HI were added to the upstream and downstream ends of the signal peptide, respectively. The signal peptide was then digested with *Xba*I and *Bam*HI and fused in front of *G2 aroA* to construct the plant transformation vector p3301-121spG2. The *G2 aroA* in vector p3301-121spG2 was replaced with *HTG7 aroA*, *AM79 aroA*, *A1501 aroA* and *RD aroA* to obtain the vectors p3301-121spHTG7, p3301-121spAM79, p3301-121spA1501 and p3301-121spRD, respectively. All plasmids used in the research are listed in Table S2.

### Evaluation of glyphosate tolerance in *E. coli*

pACYC184, pACYC-HTG7, pACYC-AM79, pACYC-A1501, pACYC-RD and pACYC-G2 were transformed into *E. coli* ER2799 competent cells and plated on LB solid medium with 50 µg mL^−1^ tetracycline. The positive clones were identified using PCR amplification of the *aroA* genes. The single clone was inoculated in 5 mL LB liquid medium and grown overnight at 37°C until the OD_600_ reached approximately 0.6. All of the cultures were centrifuged, and the pellets were resuspended to OD_600_ = 0.5 with M9 liquid basic medium. Then the 500 µL cultures were subcultured to 200 mL M9 liquid medium containing 0, 20, 100, 150 and 200 mM glyphosate. The absorbance at OD_600_ was measured at 6, 8, 10, 12, 14, 16, 18, 20 and 22 h. The experiment was repeated three times. The empty strain ER2799 was detected as a control.

### Enzyme assay

Single colony of the transformant was grown overnight in LB medium with 100 µg mL^−1^ kanamycin and 25 µg mL^−1^ chloromycetin at 37°C. 1 mL of overnight culture was added into 100 mL LB medium and grew to OD_600_ = 0.4, then 0.5 mM isopropyl β-D-thiogalactoside (IPTG) was added and the cells were incubated for another 4 h at 28°C or for 12 h at 16°C. The proteins were purified using Ni^2+^-NTA spin column (Qiagen) according to the described protocol. Protein samples were analyzed by SDS-PAGE electrophoresis.

The activity of EPSPS was assayed at 25°C in a 20 μl mixtures containing 50 mM HEPES-NaOH (pH 7.0), 2 mM dithiothreitol, 100 mM KCl, 1 mM S3P and varied concentrations of glyphosate and PEP [4], [42].The reaction was initiated by the addition of enzyme (19 μg mL^−1^ HTG7 EPSPS, 19.2 μg mL^−1^ AM79 EPSPS, 21.2 μg mL^−1^ A1501 EPSPS, 8.08 μg mL^−1^ RD EPSPS, 26.4 μg mL^−1^ G2 EPSPS) and allowed to proceed for 3 min before the addition of 160 μl Lanzetta reagent. Color development was stopped after 3 min by adding 20 μl of 34% (w/v) sodium citrate, The change in absorbance at 655 nm was recorded 20 min later, and the amount of inorganic phosphate was calculated by comparison with phosphate standards. Enzymatic activity is expressed as increased amount of Pi (mmol) (min reaction time)^−1^ (mg protein)^−1^ (U mg^−1^).The *K*m values for PEP were determined by fitting data to Michealis equation *V* = *V*_max_ [*S*]/*K*_m_+[*S*], where *V* is the velocity of the reaction, *V*_max_ is the maximum velocity, [*S*] is the substrate(PEP) concentration, *K*_m_ is the Michaelis constant. The *K*_i_ is derived by determining the *K*_m(obs)_ of PEP in the presence of increasing concentration of glyphosate. The date was fitting to the equation *K*_m(obs)_ = (*K*_m_/*K*_i_)[*I*]+*K*_m_, where *K*_m(obs)_ is the Michaelis constant for PEP in the presence of glyphosate, [*I*] is the concentration of glyphosate, and *K*_m_ is the Michaeliss constant for PEP in the abence of glyphosate.

### Tobacco transformation

Plant expression plasmids p3301-121spG2, p3301-121spHTG7, p3301-121spAM79, p3301-121spA1501 and p3301-121spRD were transferred into competent cells of the *A. tumefaciens* strain LBA4404 through freeze-thaw treatment. The transformed *A. tumefaciens* colonies were selected on YEB-agar plates containing 100 µg mL^−1^ of kanamycin and 125 µg mL^−1^ of streptomycin. The positive colonies were identified by PCR amplification of the inserted genes and used for the tobacco transformation as previously described [43] using phosphinothricin (PPT) as the selecting gene. The transgenic plants were confirmed by PCR amplification of the *aroA* gene.

### Southern blot analysis

Genomic DNA was isolated from young leaves of tobacco plants using the CTAB method. 100 µg genomic DNA was digested by *Hin*dIII, which has only one recognition site within the plasmid, electrophoresed on 1% (w/v) agarose gel and transferred onto a Hybond^+^ nylon membranes (Roche, Mannheim, Germany). The PCR fragment of the *aroA* gene was amplified using primers (Table S3), and was labeled using a DIG High Prime DNA Labeling and Detection Starter Kit II (Roche, Mannheim, Germany), and hybridization and detection steps were performed according to the manufacturer's instructions.

### RNA isolation and quantitative real-time RT-PCR

Total RNA from *E. coli* and tobacco leaves was isolated using Trizol reagent (Invitrogen, Carlsbad, CA, USA) and first-strand cDNA synthesis was performed with M-MLV reverse transcriptase (Promega) using oligo-dT primer. Specific primer for each gene was shown in Table S3. For quantitative real-time RT-PCR, 1 µl cDNA was mixed with 2×SYBR premix ExTaq (Takara), 0.2 µM forward primer, 0.2 µM reverse primer and 0.4 µl 50×ROX in 20 µl reaction mixture. Quantitative real-time RT-PCR was done using ABI 7300 system with the following protocol: 95°C 2 min, 40 cycles for 95°C 5 s, 58°C 30 s, 72°C 31 s. The relative transcript level was calculated using the 2^−ΔΔ^Ct method with tobacco actin gene or bacterial 16S rRNA gene as housekeeping genes.

### Glyphosate tolerance analysis of transgenic plants

Heterozygous tobacco seeds of the T1 generation were germinated on MS medium containing 10 mg L^−1^ PPT and grown for 7 days at 100 µmol s^−1^ m^−2^ with a 16 h light/8 h dark period. The living seedlings with a similar size were transferred to MS medium in plates containing different amounts of glyphosate and grown vertically for another two weeks. Injury was observed, and the fresh weight was measured. One-week-old seedlings were transferred to soil and grown in a greenhouse for one month. Six-to-eight-leaf stage transgenic plants were sprayed with 6 L ha^−1^ Roundup®, injury was observed and survival rate was measured 2 weeks after treatment.

### Shikimate measurement

One-month-old plants were sprayed with 0.5, 1 or 2 L ha^−1^ Roundup®. Leaf samples were taken at 0, 1, 3, 5 and 7 d after the glyphosate treatment for shikimate measurement. Approximately 0.5 g leaf was ground in liquid nitrogen, extracted in 1 mL of 0.25 N HCl, and then centrifuged at 12,000 rpm for 15 min at 4°C. The supernatant was collected and filtered through 0.45 µm nylon membrane. Shikimate concentration was determined by liquid chromatograph mass spectrometer (LC-MS) (LCMS-2010A, Shimadzu), equipped with an PTH-C18 column (250×4 mm, 5 µm)(Shimadzu, Japan) maintained at 40°C. The mobile phase was 0.1% trifluoroacetic acid (v/v) in 5% methanol, with a flow rate of 0.5 mL min^−1^ at 40°C. Shikimate was detected at 210 nm and quantified based on standard shikimate (Sigma-Aldrich).

### Data analysis

Data were analyzed using ANOVA method in computer SAS software and the results were compared by using Duncan's multiple range tests.

## Supporting Information

Figure S1 **The transcription level of** ***aroA*** **genes in** ***E. coli*** **ER2799.** *E. coli* ER2799 containing different *aroA* genes grew in M9 medium without or with 200 mM glyphosate for 16 h. Data are shown as mean *Ct*_gene_/*Ct*_16S_ value ± S.E. for three independent biology replicates.
(TIF)

Figure S2 **Enzyme kinetics analysis of five bacterial EPSPS proteins.** SDS-PAGE electrophoresis of purified proteins (A), *K*_m_ and *K*_i_ measurement of HTG7 EPSPS (B), AM79 EPSPS (C), A1501 EPSPS (D), RD EPSPS (E) and G2 EPSPS (F). The observed *K*_m_ was measured at PEP concentrations ranging from 0 to 1 mM, and was plotted against the glyphosate concentration to obtain the *K*m and *K*i for the enzyme.
(TIF)

Figure S3 **PCR analysis of transgenic tobacco plants.** PCR analysis of transgenic tobacco harboring *HTG7* (A), *AM79* (B), *A1501* (C), *RD* (D) and *G2* (E). M, DL 2000 plus DNA ladder; CK-, water as PCR control; WT, non-transgenic tobacco line; CK1, plasmid pACYC-HTG7 as a positive control; CK2, plasmid pACYC-AM79 as a positive control; CK3, plasmid pACYC-A1501 as a positive control; CK4, plasmid pACYC-RD as a positive control; CK5, plasmid pACYC-G2 as a positive control; 1–6, different transgenic tobacco lines.
(TIF)

Figure S4 **Glyphosate tolerance analysis of the transgenic tobacco expressing** ***G2 aroA*** **with or without rbcS signal peptide.** Photograph of four-to six-leave stage tobacco plants two weeks after 1 L ha^−1^ Roundup® treatment. Left, non-transgenic tobacco; middle, tobacco plants harboring plasmid p3301-121G2 without signal peptide; right, tobacco plants harboring plasmid p3301-121spG2 with signal peptide.
(TIF)

Figure S5 **The transcription level of** ***aroA*** **genes in transgenic tobacco plants.** Data are shown as mean *Ct* value ± SE for three independent biology replicates. The relative transcription level was analyzed using 2^−ΔΔCt^ method, and one line with the highest transcription level among different lines transformed with the same construct was normalized as 1.00. ^*^Different letter means significant difference at *P*<0.05 level (Duncan's multiple range tests).
(TIF)

Figure S6 **Glyphosate tolerance of** ***E. coli*** **expressing** ***AM79 aroA*** **or** ***CP4 aroA*** **.** *AM79 aroA* or *CP4 aroA* was cloned into *Bam*HI/*Sa*lI site of plasmid pACYC184. plasmids were transformed into *E. coli* ER2799 competent cells. M9 liquid medium was supplemented with different concentrations of glyphosate. OD_600_ was recorded 16 h after treatment. Data are shown as the average ± S.E. of three independent experiments.
(TIF)

Figure S7 **Amino acid alignment of the EPSPS proteins and key active site residues analysis.** Amino acid alignment of the five EPSPS proteins used in this study and the other three EPSPS proteins from *E. coli*, *Agrobacterium* sp. CP4 and *Streptococcus pneumoniae*. The classical key active sites residues of known EPSPS structures are marked using black dots. One dot indicates the universal active site, two dots show that the active sites are special in some amino acids and three dots indicate that the active site is unique in the amino acid sequence. The red frame marked I, II, III, IV, V indicates the five conserved domains in class II EPSPS enzymes. The underlined region indicates the general conserved domain in class I EPSPS enzymes.
(TIF)

Table S1 **Oligonucleotides used for vector construction in this study.**
(DOC)

Table S2 **Strains and plasmids used in this study.**
(DOC)

Table S3 **Oligonucleotides used for Real-time RT-PCR and Southern blot analysis.**
(DOC)
