# Supplementary figures and images for: A Novel 5-Enolpyruvylshikimate-3-Phosphate Synthase Shows High Glyphosate Tolerance in Escherichia coli and Tobacco Plants
Source: PLoS One. 2012 Jun 8;7(6):e38718. doi: 10.1371/journal.pone.0038718 (PMC3371024; doi:10.1371/journal.pone.0038718)

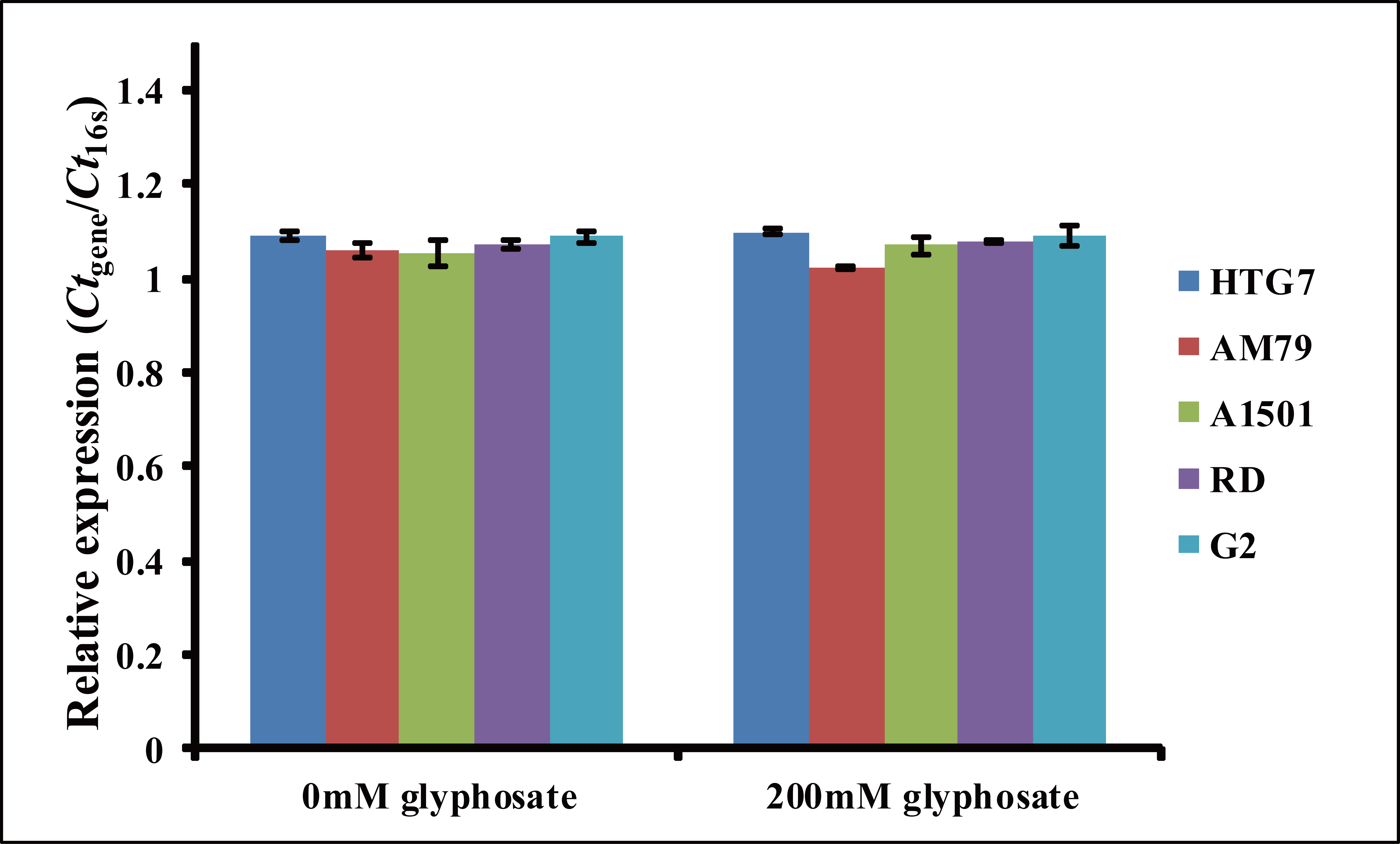

Supplement: Figure S1 — The transcription level of aroA genes in E. coli ER2799. E. coli ER2799 containing different aroA genes grew in M9 medium without or with 200 mM glyphosate for 16 h. Data are shown as mean Ct gene/Ct 16S value ± S.E. for three independent biology replicates. (TIF) [file pone.0038718.s001.tif]

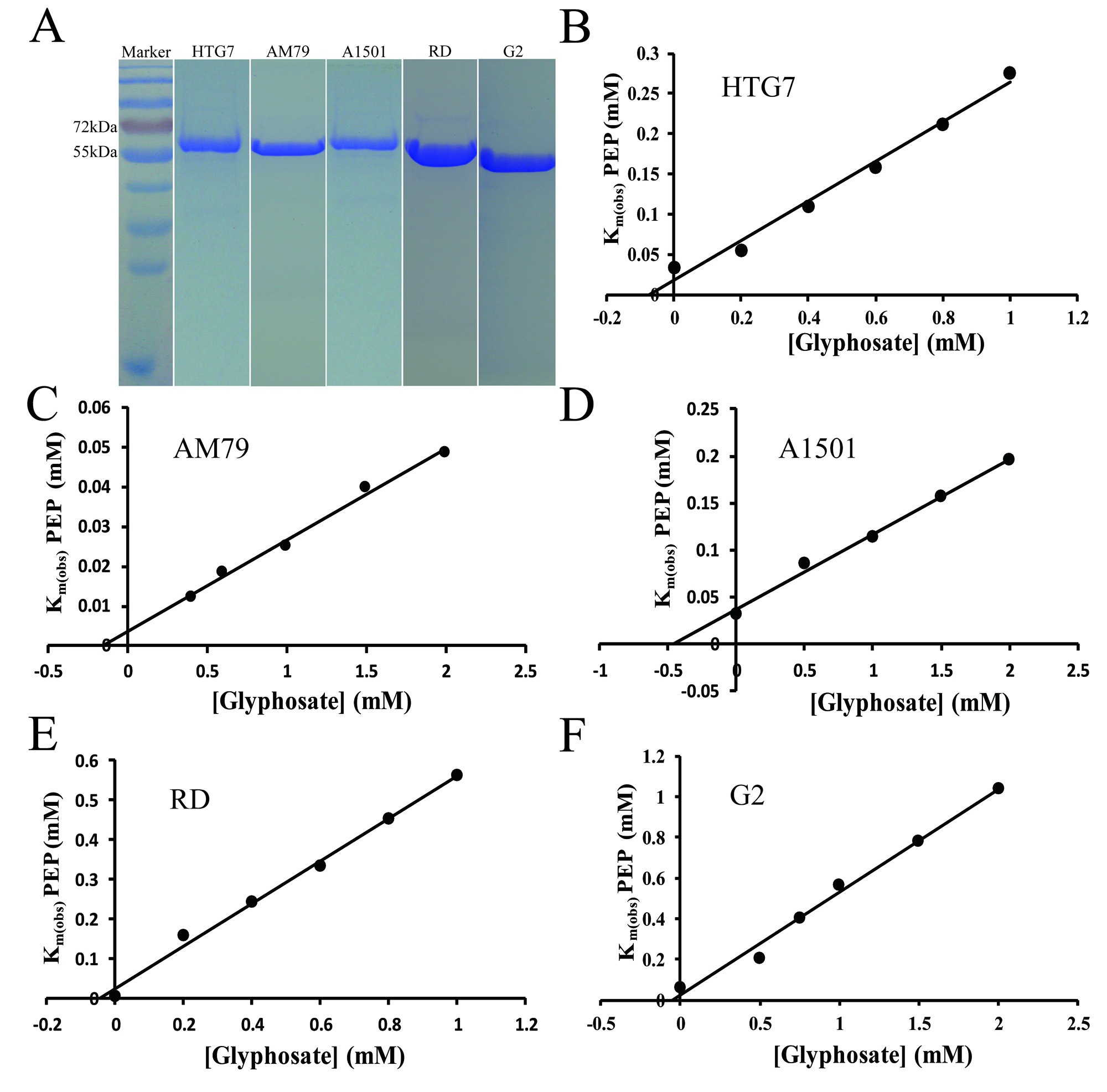

Supplement: Figure S2 — Enzyme kinetics analysis of five bacterial EPSPS proteins. SDS-PAGE electrophoresis of purified proteins (A), K m and K i measurement of HTG7 EPSPS (B), AM79 EPSPS (C), A1501 EPSPS (D), RD EPSPS (E) and G2 EPSPS (F). The observed K m was measured at PEP concentrations ranging from 0 to 1 mM, and was plotted against the glyphosate concentration to obtain the Km and Ki for the enzyme. (TIF) [file pone.0038718.s002.tif]

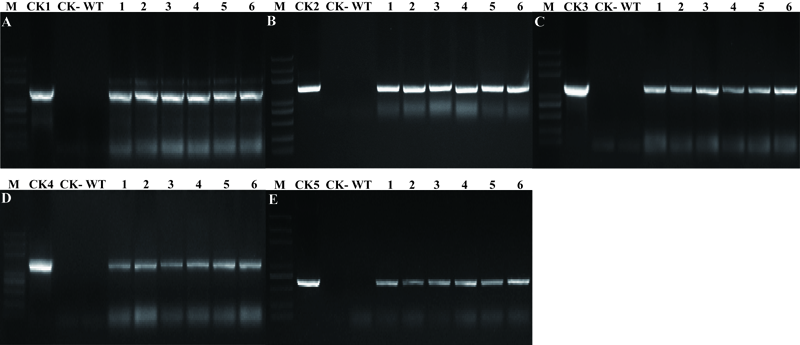

Supplement: Figure S3 — PCR analysis of transgenic tobacco plants. PCR analysis of transgenic tobacco harboring HTG7 (A), AM79 (B), A1501 (C), RD (D) and G2 (E). M, DL 2000 plus DNA ladder; CK-, water as PCR control; WT, non-transgenic tobacco line; CK1, plasmid pACYC-HTG7 as a positive control; CK2, plasmid pACYC-AM79 as a positive control; CK3, plasmid pACYC-A1501 as a positive control; CK4, plasmid pACYC-RD as a positive control; CK5, plasmid pACYC-G2 as a positive control; 1–6, different transgenic tobacco lines. (TIF) [file pone.0038718.s003.tif]

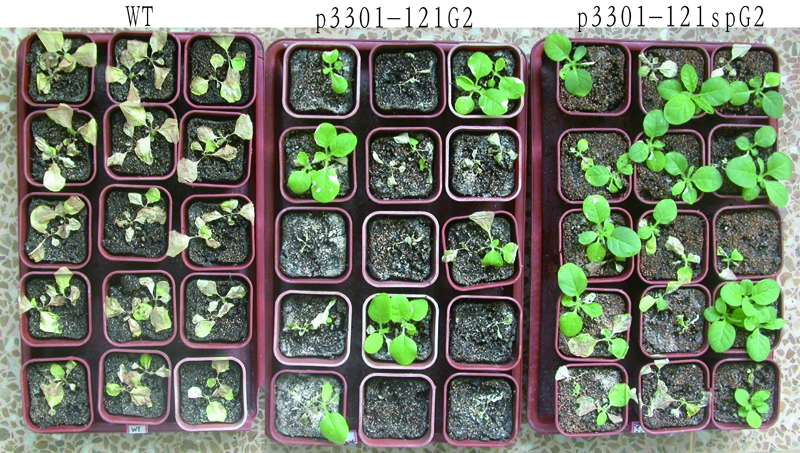

Supplement: Figure S4 — Glyphosate tolerance analysis of the transgenic tobacco expressing G2 aroA with or without rbcS signal peptide. Photograph of four-to six-leave stage tobacco plants two weeks after 1 L ha−1 Roundup® treatment. Left, non-transgenic tobacco; middle, tobacco plants harboring plasmid p3301-121G2 without signal peptide; right, tobacco plants harboring plasmid p3301-121spG2 with signal peptide. (TIF) [file pone.0038718.s004.tif]

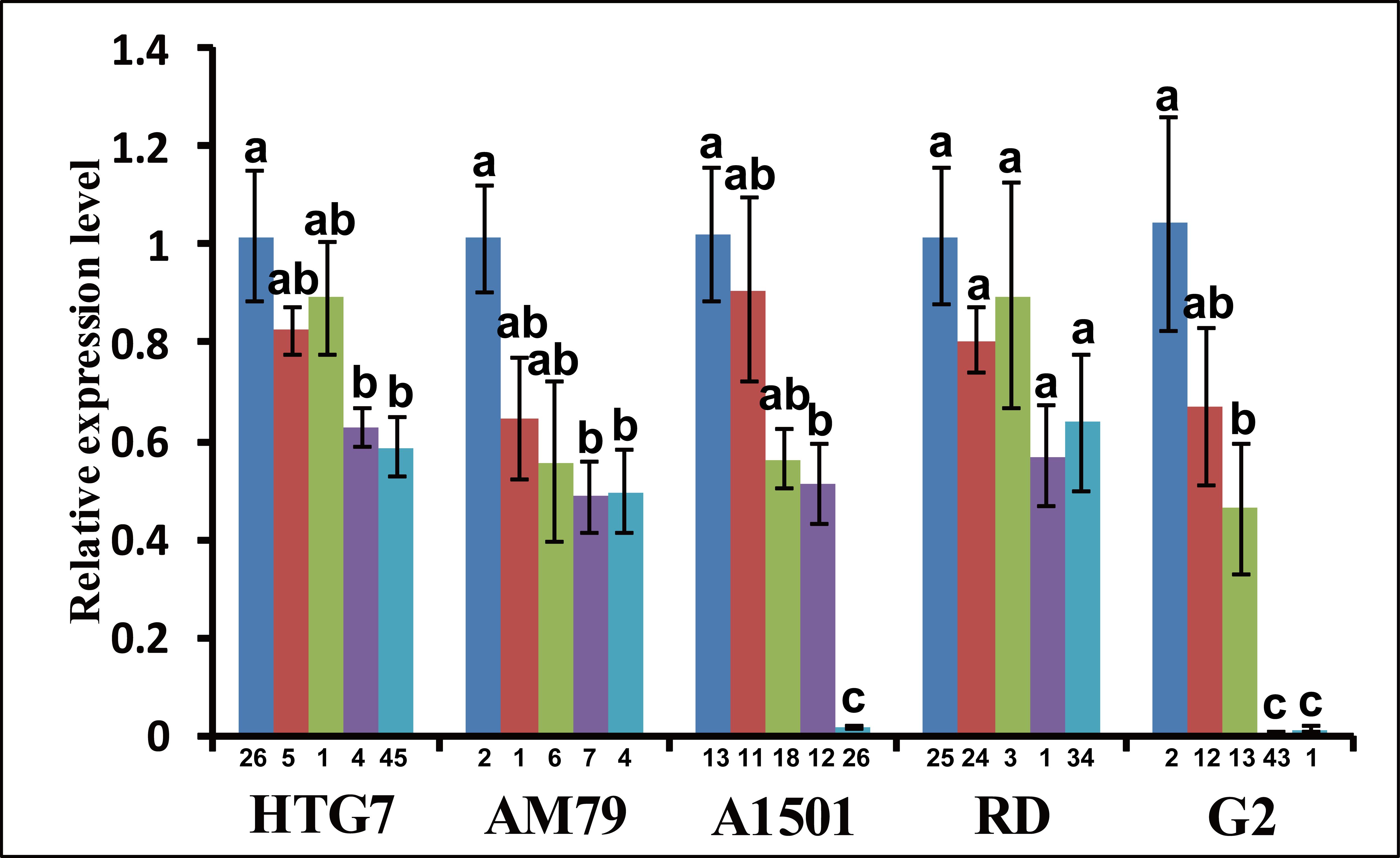

Supplement: Figure S5 — The transcription level of aroA genes in transgenic tobacco plants. Data are shown as mean Ct value ± SE for three independent biology replicates. The relative transcription level was analyzed using 2−ΔΔCt method, and one line with the highest transcription level among different lines transformed with the same construct was normalized as 1.00. *Different letter means significant difference at P<0.05 level (Duncan's multiple range tests). (TIF) [file pone.0038718.s005.tif]

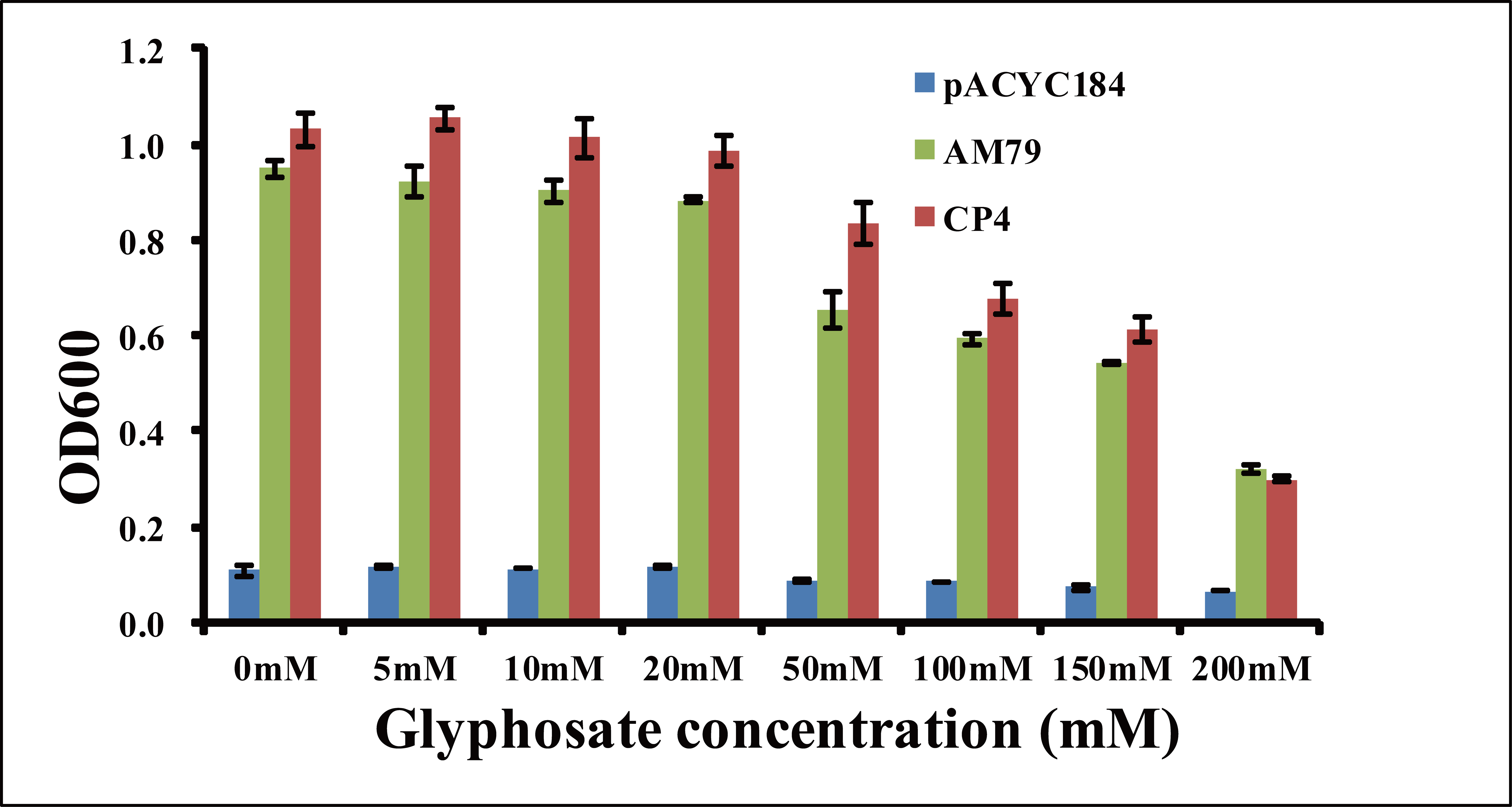

Supplement: Figure S6 — Glyphosate tolerance of E. coli expressing AM79 aroA or CP4 aroA . AM79 aroA or CP4 aroA was cloned into BamHI/SalI site of plasmid pACYC184. plasmids were transformed into E. coli ER2799 competent cells. M9 liquid medium was supplemented with different concentrations of glyphosate. OD600 was recorded 16 h after treatment. Data are shown as the average ± S.E. of three independent experiments. (TIF) [file pone.0038718.s006.tif]

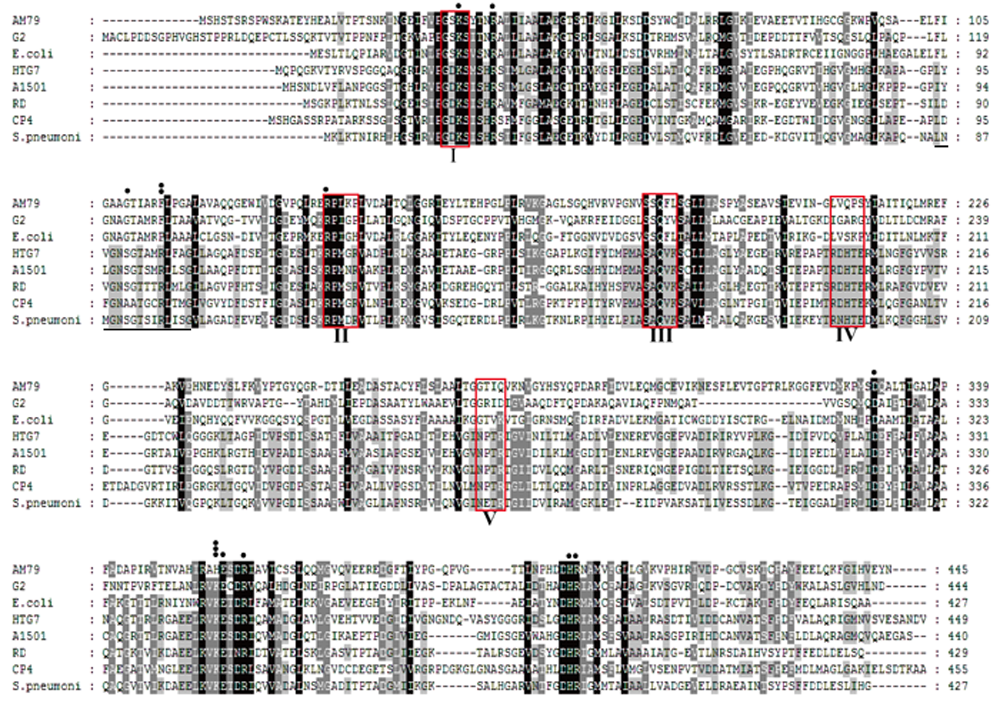

Supplement: Figure S7 — Amino acid alignment of the EPSPS proteins and key active site residues analysis. Amino acid alignment of the five EPSPS proteins used in this study and the other three EPSPS proteins from E. coli, Agrobacterium sp. CP4 and Streptococcus pneumoniae. The classical key active sites residues of known EPSPS structures are marked using black dots. One dot indicates the universal active site, two dots show that the active sites are special in some amino acids and three dots indicate that the active site is unique in the amino acid sequence. The red frame marked I, II, III, IV, V indicates the five conserved domains in class II EPSPS enzymes. The underlined region indicates the general conserved domain in class I EPSPS enzymes. (TIF) [file pone.0038718.s007.tif]
