# Supplementary material for: A Novel 5-Enolpyruvylshikimate-3-Phosphate Synthase Shows High Glyphosate Tolerance in Escherichia coli and Tobacco Plants
Source: PLoS One. 2012 Jun 8;7(6):e38718. doi: 10.1371/journal.pone.0038718 (PMC3371024; doi:10.1371/journal.pone.0038718)
Supplement: Table S1 — Oligonucleotides used for vector construction in this study. (DOC) [file pone.0038718.s008.doc]

**Table S1. Oligonucleotides used for vector construction in this study**

| name | sequence |
| --- | --- |
| E-HTG7-E-S | AAG*GAATTC*ATGCAACCACAGGGTAAA |
| E-HTG7-E-A | CCG*GAATTC*TCAAACGTCATTAGCACT |
| E-AM79-E-S | AAG*GAATTC*ATGTCACATTCTACCTCT |
| E-AM79-E-A | CCG*GAATTC*TTAATTATACTCCACATG |
| E-A1501-E-S | AAG*GAATTC*ATGCATTCCAATGACCTC |
| E-A1501-E-A | ATA*GAATTC*TCATGAGGCGCCCTCC |
| E-RD-E-S | AAG*GAATTC*ATGAGTGGAAAACCATTA |
| E-RD-E-A | GCA*GAATTC*TTATTGGCTTAGCTCATC |
| E-G2-E-S | AAG*GAATTC*ATGGCGTGTTTGCCTGAT |
| E-G2-E-A | CCG*GAATTC*TCAGTCGTTTAGGTGAAC |
| B-HTG7-S-S | AA*GGATCC*ATGCAACCACAGGG |
| B-HTG7-S-A | TT*GAGCTC*TCAAACGTCATTAGC |
| B-AM79-S-S | AAT*GGATCC*ATGTCACATTCTACCTCT |
| B-AM79-S-A | CG*GAGCTC*TTAATTATACTCCACATG |
| B-A1501-S-S | AAG*GGATCC*ATGCATTCCAATG |
| B-A1501-S-A | TA*GAGCTC*TCATGAGGCGCC |
| B-RD-S-S | AAG*GGATCC*ATGAGTGGAAAACCATTA |
| B-RD-S-A | GCA*GAGCTC*TTATTGGCTTAGCTCATC |
| B-G2-S-S | AA*GGGATC*CATGGCGTGTTTG |
| B-G2-S-A | AA*GAGCTC*TCAGTCGTTTAGGTGAAC |
| BamHI-AM79-SalI-S | TGT*GGATCC*GTATGTCACATTCTA |
| BamHI-AM79-SalI-A | TGGAGCGT*GTCGAC*TTAATTATACT |
| BamHI-CP4-SalI-S | C*GGATCC*ATATGGCACAAATTAACAACAT |
| BamHI-CP4-SalI-A | TT*GTCGAC*TCAGGCAGCCTTCGTAT |
| PET-HTG7-BamHI | GC*GGATCC*ATGCAACCACAGGGTAAAGTTA |
| PET-HTG7-HindIII | GC*AAGCTT*GAACGTCATTAGCACTCTCCACA |
| PET-AM79-BamHI | GC*GGATCC*ATGTCACATTCTACCTCTAGGTCC |
| PET-AM79-HindIII | GC*AAGCTT*GATTATACTCCACATGTATTCCAAA |
| PET-A1501-BamHI | GC*GGATCC*ATATGCATTCCAATGACCTCGT |
| PET-A1501-HindIII | GC*AAGCT*TGTGAGGCGCCCTCCGC |
| PET-RD-BamHI | GC*GGATCC*ATGAGTGGAAAACCATTAAAAACGA |
| PET-RD-HindIII | GC*AAGCTT*GTTGGCTTAGCTCATCAAGATCTT |
| PET-G2-BamHI | GC*GGATCC*ATGGCGTGTTTGCCTGATGATT |
| PET-G2-HindIII | GC*AAGCTT*GGTCGTTTAGGTGAACGCCCA |

New restriction sites are underlined and italicized.
