# Supplementary material for: A Novel 5-Enolpyruvylshikimate-3-Phosphate Synthase Shows High Glyphosate Tolerance in Escherichia coli and Tobacco Plants
Source: PLoS One. 2012 Jun 8;7(6):e38718. doi: 10.1371/journal.pone.0038718 (PMC3371024; doi:10.1371/journal.pone.0038718)
Supplement: Table S2 — Strains and plasmids used in this study. (DOC) [file pone.0038718.s009.doc]

**Table S2.** **Strains and plasmids used in this study**

| Strains or plasmids | Description | Antibiotic Resistance^a^ | Resources |
| --- | --- | --- | --- |
| ER2799 | *Escherichia coli* strain  (with the *EPSPS* deleted in genome) |  | Ref. 41 |
| Rosetta(DE3) | *Escherichia coli* expression strain | Chl | Novagen |
| pACYC184 | Cloning vector | Tet,Chl | New England Biolabs |
| pACYC-HTG7 | pACYC184 with *HTG7 aroA* in *Eco*RI | Tet | This work |
| pACYC-AM79 | pACYC184 with *AM79 aroA* in *Eco*RI | Tet | This work |
| pACYC-A1501 | pACYC184 with *A1501 aroA* in *Eco*RI | Tet | This work |
| pACYC-RD | pACYC184 with *RD aroA* in *Eco*RI | Tet | This work |
| pACYC-G2 | pACYC184 with *G2 aroA* in *Eco*RI | Tet | This work |
| p3301-121G2 | Plant expressing vector containing  *G2 aroA* without signal peptide | Kan | This work |
| p3301-121spG2 | Plant expressing vector containing  *G2 aroA* with signal peptide | Kan | This work |
| p3301-121spHTG7 | Plant expressing vector containing  *HTG7 aroA* with signal peptide | Kan | This work |
| p3301-121spAM79 | Plant expressing vector containing  *AM79 aroA* with signal peptide | Kan | This work |
| p3301-121spA1501 | Plant expressing vector containing  *A1501 aroA S* with signal peptide | Kan | This work |
| p3301-121spRD | Plant expressing vector containing  *RD aroA* with signal peptide | Kan | This work |
| pACYC-BAM79S | pACYC184 with *AM79 aroA*  between *Bam*HI and *Sa*lI | Chl | This work |
| pACYC-BCP4S | pACYC184 with *CP4 aroA*  between *Bam*HI and *Sa*lI | Chl | This work |
| pET-28a | Expression vector | Kan | Novagen |
| pET-HTG7 | pET-28a with *HTG7 aroA* between *Bam*HI and *Hin*dIII | Kan | This work |
| pET-AM79 | pET-28a with *AM79 aroA* between *Bam*HI and *Hin*dIII | Kan | This work |
| pET-A1501 | pET-28a with *A1501 aroA* between *Bam*HI and *Hin*dIII | Kan | This work |
| pET-RD | pET-28a with *RD aroA* between *Bam*HI and *Hin*dIII | Kan | This work |
| pET-G2 | pET-28a with *G2 aroA* between *Bam*HI and *Hin*dIII | Kan | This work |

^a^ Amp, ampicillin; Kan, kanamycin; Rif, rifampicin; Tet, tetracycline; Chl, chloromycetin
