# Supplementary material for: A Novel 5-Enolpyruvylshikimate-3-Phosphate Synthase Shows High Glyphosate Tolerance in Escherichia coli and Tobacco Plants
Source: PLoS One. 2012 Jun 8;7(6):e38718. doi: 10.1371/journal.pone.0038718 (PMC3371024; doi:10.1371/journal.pone.0038718)
Supplement: Table S3 — Oligonucleotides used for Real-time RT-PCR and Southern blot analysis. (DOC) [file pone.0038718.s010.doc]

**Table S3. Oligonucleotides used for Real-time RT-PCR and Southern blot analysis**

|  | Name | Sequence |
| --- | --- | --- |
| 1 | RT-HTG7-S | CATTCGCTATGTGCCTCTGA |
| 2 | RT-HTG7-A | TATGCTCCACTCCCAAAACC |
| 3 | RT-AM79-S | CACAACTGCGAGAAAGACCA |
| 4 | RT-AM79-A | GACATTTCCTGGCACCCTTA |
| 5 | RT-A1501-S | ATTCCAATGACCTCGTTTTCCT |
| 6 | RT-A1501-A | CTTCGAGAAAACCCTCGACTT |
| 7 | RT-RD-S | GCGCATTTGGAGTAGACGTAG |
| 8 | RT-RD-A | AGGATTGAGTCCGACGTTTTT |
| 9 | RT-G2-S | GCGTGTTTGCCTGATGAT |
| 10 | RT-G2-A | GGAAGTTGGGCGGTGTAA |
| 11 | RT-NtActin-S | AAGGGATGCGAGGATGGA |
| 12 | RT-NtActin-A | CAAGGAAATCACCGCTTTGG |
| 13 | RT-16srRNA-S | CGAAGAACCTTACCTGGTCTTG |
| 14 | RT-16srRNA-A | ACTGGCAGTCTCCTTTGAGTTC |
| 15 | HTG7-probe-S | GATAAATCCATGTCTCACCGTTCC |
| 16 | HTG7-probe-A | ACTAGCCATCGGCATATCGTAAAA |
| 17 | AM79-probe-S | ATGCTTCAACAGCCTGCTATTTTC |
| 18 | AM79-probe-A | TACACAACCCGGATCGACTATTCT |
| 19 | A1501-probe-S | ATTCCAATGACCTCGTTTTCC |
| 20 | A1501-probe-A | CTGGATACGATCCGACTCCTT |
| 21 | RD-probe-S | TAAAAGAGAAGGCGAGTATGTTGA |
| 22 | RD-probe-A | AAAGTAGGGTAGGAGACGTGAATG |
| 23 | G2-probe-S | GCTCCAAATCCATTACCAACC |
| 24 | G2-probe-A | CCAGGGCTTTCCAGTAGTCA |

1-14: Oligonucleotides used for Real-time RT-PCR

15-24: Oligonucleotides used Southern blot analysis
